# Exploring the effect of context and expertise on attention: is attention shifted by information in medical images?

**DOI:** 10.3758/s13414-019-01695-7

**Published:** 2019-03-01

**Authors:** Ann J. Carrigan, Kim M. Curby, Denise Moerel, Anina N. Rich

**Affiliations:** 1 Perception in Action Research Centre, Macquarie University, Sydney, NSW Australia; 2 Centre for Elite Performance, Expertise and Training, Macquarie University, Sydney, NSW Australia; 3 ARC Centre of Excellence in Cognition and Its Disorders, Macquarie University, Sydney, NSW Australia; 4 Department of Psychology, Macquarie University, Sydney, NSW Australia; 5 Department of Cognitive Science, Macquarie University, Sydney, NSW Australia

**Keywords:** Visual attention, Medical image perception, Spatial attention cueing

## Abstract

Radiologists make critical decisions based on searching and interpreting medical images. The probability of a lung nodule differs across anatomical regions within the chest, raising the possibility that radiologists might have a prior expectation that creates an attentional bias. The development of expertise is also thought to cause “tuning” to relevant features, allowing radiologists to become faster and more accurate at detecting potential masses within their domain of expertise. Here, we tested both radiologists and control participants with a novel attentional-cueing paradigm to investigate whether the deployment of attention was affected (1) by a context that might invoke prior knowledge for experts, (2) by a nodule localized either on the same or on opposite sides as a subsequent target, and (3) by inversion of the nodule-present chest radiographs, to assess the orientation specificity of any effects. The participants also performed a nodule detection task to verify that our presentation duration was sufficient to extract diagnostic information. We saw no evidence of priors triggered by a normal chest radiograph cue affecting attention. When the cue was an upright abnormal chest radiograph, radiologists were faster when the lateralised nodule and the subsequent target appeared at the same rather than at opposite locations, suggesting attention was captured by the nodule. The opposite pattern was present for inverted images. We saw no evidence of cueing for control participants in any condition, which suggests that radiologists are indeed more sensitive to visual features that are not perceived as salient by naïve observers.

**Electronic supplementary material:**

The online version of this article (10.3758/s13414-019-01695-7) contains supplementary material, which is available to authorized users.

## Introduction

The complex visual search task confronting radiologists is one that is crucial for successful cancer treatment. Early detection has been linked to better survival rates (Etzioni et al., 2003), and so understanding the way in which knowledge influences the perception of these images is an important endeavour. Statistically, certain pathologies occur more frequently in specific locations. For example, in the lung, primary malignant nodules are one and a half times more likely to occur in the right lung than in the left, and mostly in the upper lobe (Garland, 1961; Swensen et al., 2000; Winer-Muram et al., 2002). Disease of the lung includes primary cancers as well as single pulmonary nodules. Nodules have a detection rate of .09% to 7% on routine chest radiographs and have been identified in 7% of 1,000 healthy volunteers in chest screening radiographs (Patel et al., 2013). They are seen in around 20% of lung cancer screening studies that use low-dose chest computer tomography (Bach et al., 2012). Although these nodules are often benign when small, they are at risk of developing into malignant disease (Midthun, Swensen, & Jett, 1993), making them a key target for radiologists during screening. Localised lung cancer (constrained to the lung) has a 5-year survival rate of 56%, and only 5% when the cancer involves other organs (American Lung Association, 2018). Thus, nodules are clinically significant and important in a screening environment. Experienced radiologists are therefore exposed to nodules, most frequently in the upper right lung, in their daily work. This makes chest radiographs containing nodules ideal stimuli to test the hypothesis that experience with statistical regularities across stimuli affects the way in which medical images are examined.

Targets of a visual search in the real world, whether a nodule in a lung or a weapon in a bag, sit in relation to surrounding objects that give them global context (Biederman, 1972). Laboratory-based studies (using simple arrays) have shown that participants are sensitive to the global context and the statistical regularities in a display. This type of incidental statistical learning has been shown to affect the allocation of spatial attention within visual search displays, referred to as contextual cueing (Chun & Jiang, 1998; Jiang, Swallow, & Rosenbaum, 2013). In a series of experiments, Chun and Jiang (1998) presented different spatial layouts (global context) of objects (Ts) among distractors (Ls). The task was to discriminate the orientation of the target T. Half of the layouts were repeated across the experimental blocks in which the target object location (but not orientation) remained constant. Participants were unaware of the repetition. The results showed that when the display was repeated, targets were discriminated more quickly, meaning that the context of the target was implicitly learned during the experiment. Recent work has shown that spatial reaction time (RT) for locating targets is faster in high probability compared to low probability locations (Jiang, 2018). This type of learning has been described as location probability learning (LPL), which implicitly induces a generalized spatial preference (Jiang, Li, & Sisk, 2018). Using more naturalistic stimuli (natural scenes), Brockmole and Henderson (2006) showed increased recall for target positions for repeated scenes. One explanation of contextual cueing is that the repeated context results in attention being guided to the target location more efficiently. Others suggest that the repeated context instead facilitates response selection (Kunar, Flusberg, Horowitz, & Wolfe, 2007). For radiologists, the global context of a medical image may well form a type of contextual cue, in the sense that there are clear background characteristics that are present in each radiograph that may invoke certain search patterns or attentional shifts. Previous studies have demonstrated that background statistics such as texture and color can indeed influence target detection (Kunar, John, & Sweetman, 2014; Kunar, Flusberg, & Wolfe, 2006). Thus, presenting an irrelevant medical image could act as a context and therefore influence subsequent performance.

An effect on attention by the presentation of a chest radiograph might be akin to what has been previously described in the natural scene literature as “scene-based guidance” (Torralba, Oliva, Castelhano, & Henderson, 2006; Wolfe, Võ, Evans, & Greene, 2011). This guidance is thought to result from the build-up of a cognitive representation of how specific scenes appear (e.g., a kitchen), and how the items contained within it are spatially represented. It is influenced by scene structure and the sum of our past experiences. In a chest radiograph, this scene structure might include the structure of the lung, the cardiac shadow, the stomach bubble, and the most likely location and features of potential abnormalities. For those with experience reading medical images, such image-based guidance, or priors, might similarly guide attention in the context of their medical expertise.

One way to test whether a stimulus produces a shift of attention is to look at its effect on performance of a subsequent task. In classic spatial-orienting experiments (e.g., Posner, 1980), participants are asked to detect a visual target presented at a left or right peripheral location. On each trial, a cue or prime stimulus appears prior to the target display. In exogenous cueing paradigms, the non-predictive cue appears in either the same location as the subsequent target (valid; 50% of trials) or in the opposite location (invalid; 50% of trials). When a cue is salient, it involuntarily captures attention to its location (Jonides, 1981), causing a measurable effect on the response to the target (a cueing effect: valid reaction time (RT) < invalid RT). Here, we tested radiologists and age- and sex-matched control participants on a novel modification of the classic Posner cueing paradigm (1980) where chest radiographs were used as cues. This allowed us to explore the influence of radiology expertise on the allocation of attention within these images.

Our first aim was to test whether the context of a medical image would result in an attentional bias in radiologists to the region of a chest radiograph most likely to contain a nodule (upper right quadrant). If the presentation of a medical image, regardless of whether it contains a nodule or not, provides a context that activates attentional priors regarding nodule likelihood, radiologists presented with normal chest radiographs may show faster responses to subsequent targets appearing on the side most likely to show nodules (right) than to targets appearing at other locations. We tested this in our first blocked condition, predicting that this contextual cue would bias attention towards the right lung.

Our second aim was to test whether expertise boosts the salience of subtle signals. Radiologists can detect abnormalities in briefly presented displays (Carrigan, Wardle, & Rich, 2018; Evans, Georgian-Smith, Tambouret, Birdwell, & Wolfe, 2013; Evans, Haygood, Cooper, Culpan, & Wolfe, 2016; Kundel & Nodine, 1975). Nodine and Krupinski (1998) proposed that after long hours of perceptual learning, experts’ perceptual systems are selectively tuned for relevant features when performing a task. For instance, in one study, radiologists with experience in breast mammography fixated on 67% of cancers within 1 s of viewing a mammogram (Kundel, Nodine, Krupinski, & Mello-Thoms, 2008). In chest radiographs, experts viewing chest radiographs fixated only 33% of lung nodules within the first second (Donovan & Litchfield, 2013). In another study, radiologists with experience reading chest radiographs were 70% accurate in detecting abnormalities after these images are flashed for only 200 ms (Kundel & Nodine, 1975). These effects are thought to be due to the ability of specialists to recognise deviations from normal structures (or layout) rapidly, allowing them to identify abnormalities. The information extracted at this early stage is thought to be mostly based on pattern recognition, which is compared with a cognitive template of “normal” to reach a diagnostic decision (Nodine & Mello-Thoms, 2010). Indeed, in the vision literature, studies suggest the existence of “search templates,” which direct attention to objects with shared features (e.g., Chun & Jiang, 1998). If radiologists have greater sensitivity to the features (or pattern) of a nodule, they should be more sensitive (i.e., “tuned”) to these features in medical images. This could be termed an expertise-related “attentional set” (Folk, Remington, & Wright, 1994) for specific nodule features that we would not expect to see in naïve observers.

A heightened sensitivity to clinically relevant features among radiologists compared to novices should result in radiologists showing attentional capture by nodules in a chest radiograph that fail to capture attention amongst observers with no prior experience reading medical images. We tested this hypothesis in a second blocked condition in which chest radiographs containing a single subtle nodule (equiprobable in left or right lung) were presented as cues, followed by the same visual detection task as in Block 1. We predicted a cueing effect with faster reaction times when the nodule was on the same side as the subsequent target (valid trials) relative to when the nodule was on the opposite side as the subsequent target (invalid trials).

In addition to answering our primary question, an effect of the presence of a nodule within the chest radiograph on subsequent target detection would also provide an indirect measure of the degree to which localisation information is extracted from this brief exposure. Previous research suggests that brief durations are sufficient for experts to detect abnormalities (e.g., Evans et al., 2013; Kundel & Nodine, 1975), but there is debate about the extent to which they can localize abnormalities at these durations (see Carrigan et al., 2018). The presence of an attentional cueing effect for radiologists from radiographs containing lateralized nodules would indicate, first, that the signal from the nodule is sufficient to capture attention, and, second, that the processing of these brief displays contains some localization information.

We had our participants complete a questionnaire about the distribution of nodules in chest radiographs after completing the first two blocks to test their explicit knowledge of nodule location probabilities. We also had them complete a third block of trials in which they performed a nodule detection task on the images to see whether they could detect the nodule when it was task-relevant to do so. Finally, we tested whether the cueing effects were orientation and context specific by presenting the identical nodule cue task for a fourth block, but with inverted chest radiographs. Inversion maintains the lower-level perceptual features of a stimulus but has been shown to robustly disrupt many of the advantages afforded to experts through their experience such as holistic processing (e.g., Curby, Glazek, & Gauthier, 2009; Tanaka & Sengco, 1997). Expertise researchers often use inverted stimuli as a control to ensure that the lower-level perceptual features within an image, in the absence of the expert context, are not driving a particular effect. In a recent study, Chin, Evans, Wolfe, Bowen, and Tanaka (2018) tested expert mammographers and showed that inverted mammograms impaired detection recognition when compared with upright mammograms. Here, we inverted the chest radiographs to assess whether any priming effects observed were driven by low-level features of the image that remain intact with inversion or whether they are dependent on the chest radiographs being presented in the (upright) context consistent with expertise. A subset of the radiologists and all of the control participants completed the cueing task with inverted chest radiograph cues.

To pre-empt our results, normal chest radiographs by themselves do not affect attention, but when the images contain a nodule, experienced radiologists show a cueing effect on a subsequent simple visual detection task, suggesting the nodules do capture attention to their location. This effect is reversed when the chest radiographs are inverted. We interpret these results as evidence for greater sensitivity of radiologists than controls to features that indicate an abnormality, which results in attentional capture by lung nodules even when they are irrelevant to the task at hand.

## Method

### Participants

#### Radiologists

Data were collected from 28 participants who volunteered in a conference setting. This sample size is similar to that of previous medical image perception studies (e.g., Carrigan et al., 2018; Chin et al., 2018; Evans et al., 2013), and was essentially constrained by the number of available participants within the conference timeframe. Two data sets were excluded on the basis of one not being a radiologist (radiographer) and one for participant error (could not comprehend instructions) in the cueing blocks leaving 26 data sets for analysis: nine females, mean age = 47 years, *SD* = 11 years, range = 27–63 years. Of these, 20 were qualified radiologists: mean years qualified = 21 years, *SD* = 11 years, range = 9–35 years, and six were radiology residents: mean years reading medical images = 3 years, *SD* = 2 years, range = 2–6 years. All were right-handed, reported normal or corrected-to-normal vision, and were naïve to the purposes of the experiment.

The study was approved by the Macquarie University Human Research Ethics Committee (Medical Sciences).

#### Controls

**Fig. 1:**
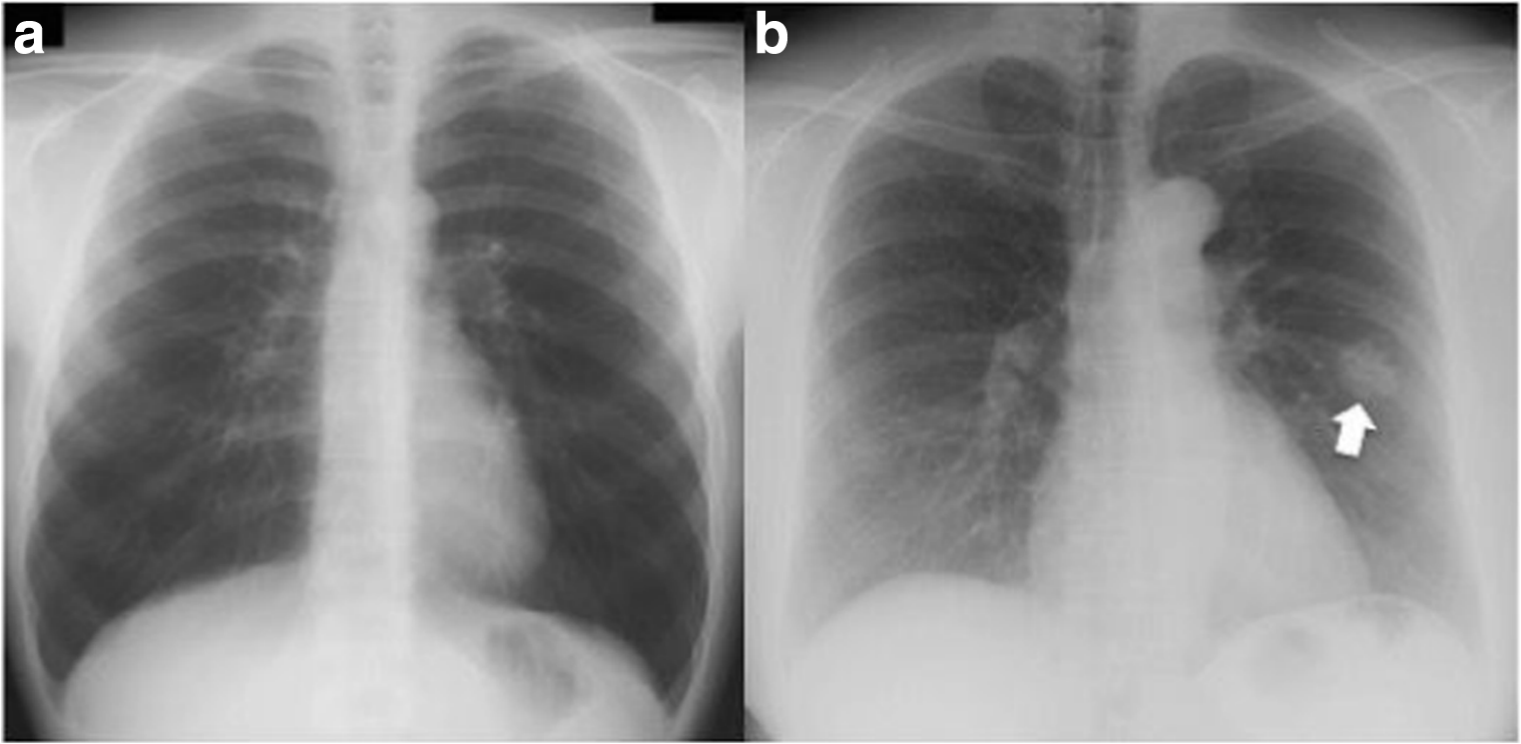
Exemplars from the stimuli sets. (**a**) Normal chest radiograph presented in the chest priors task; (**b**) nodule radiograph presented in the nodule task (indicated by the white arrow, not present in the actual displays)

Twenty-six observers matched on sex and age (±4 years) to the radiologists from the Macquarie University community (nine females, mean age = 43 years, *SD* = 10 years, range = 27–63 years) volunteered for the study. All but one were right-handed, and all reported normal or corrected-to-normal vision and were naïve to the purposes of the experiment. As the participants had no prior experience viewing chest radiographs, they were initially shown a chest image (one normal, one nodule) and familiarized with the radiological anatomy of a chest (Fig. 1).

### Stimuli and apparatus

**Fig. 2:**
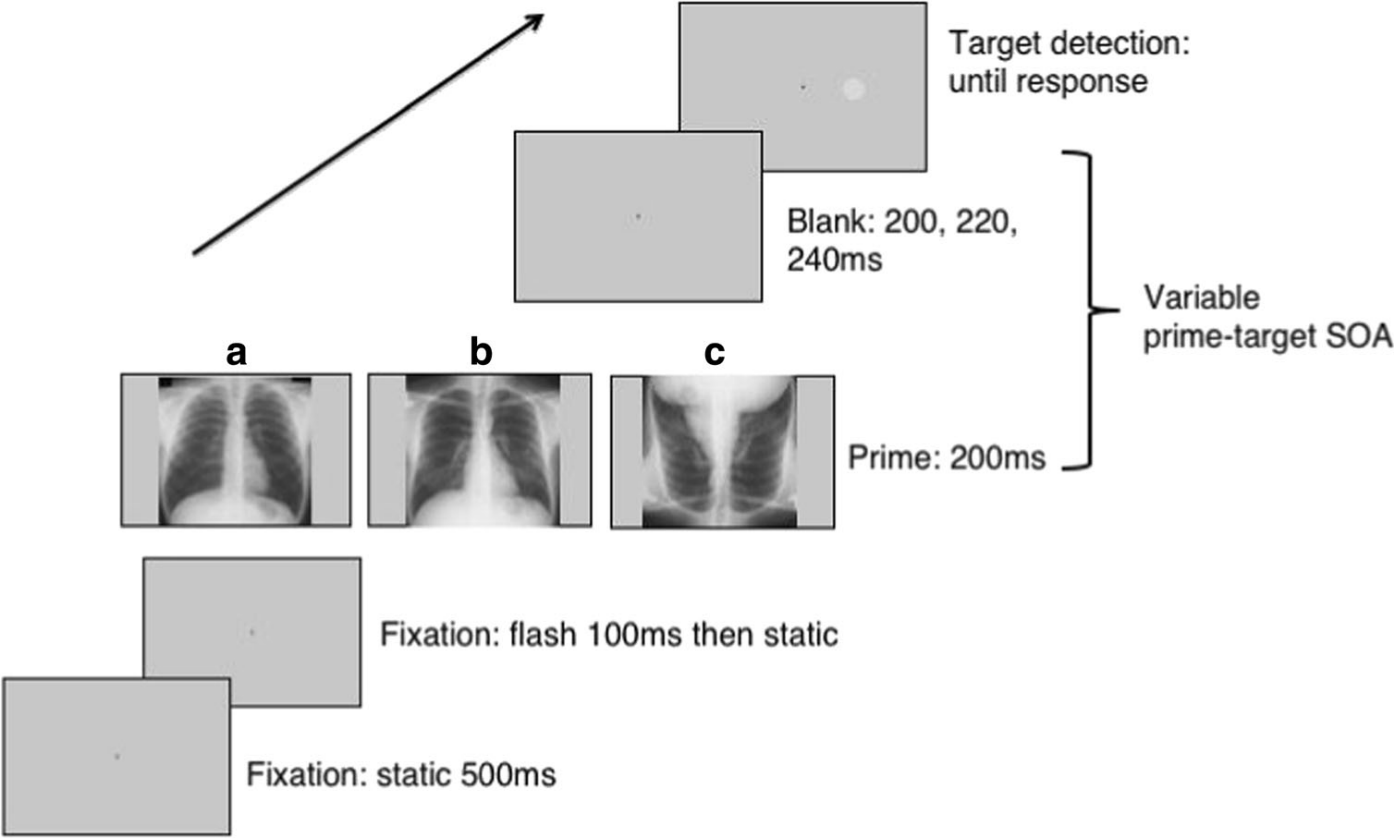
Example of an experimental trial shown to the participants. Trials began with a fixation cross followed by the prime display. In separate blocks, the prime display was either (a), (b), or (c), corresponding to our chest priors, upright nodule, and inverted nodule tasks. The prime-target SOA varied between 400, 416.7, and 450 ms for the radiologists (screen refresh = 60 Hz) and 400, 425, and 441.7 ms for the controls (screen refresh = 120 Hz). (Note: the target is made larger and brighter for illustration purposes)

The central fixation point was a cross measuring 0.5° of visual angle that appeared against a gray background (RGB triplet: 200, 200, 200), and the target was a low-contrast gray circle (RGB triplet: 195, 195, 195; 1° in diameter; see Fig. 2). The primes consisted of 124 de-identified, posterior-anterior chest radiographs (62 normal, 62 nodule), downloaded from the Japanese Society of Radiological Technology database (JSRT: Shiraishi et al., 2000), which is publicly available at http://www.jsrt.or.jp/jsrt-db/eng.php. The nodule diameters range from 8 to 37 mm (mean = 19 mm), they are located throughout the lungs (also behind the heart and under the diaphragm), and their intensities (densities) vary from nearly invisible to very bright. The nodules are subdivided in five categories, based on the degree of subtlety for detection (rated by three independent chest radiologists), which is influenced by the nodule size, occlusion by other structures, and nodule density. For the experimental trials, we balanced nodule subtlety across the left and right hemifields. There were 48 images in each image set and nodule location was balanced across lung field (50% left, 50% right).

For the radiologists, the experiment was conducted in a quiet reading-like room at a large radiology conference. The stimuli were presented on a Macintosh MacBook Pro with a separate screen using MATLAB 2011B with Psychtoolbox Version 3 (Kleiner, Brainard, & Pelli, 2007). Stimuli were downsized to 800 × 800 pixels centered on a 1,920 × 1,080 resolution, 27-in. QNIX 2710, liquid-crystal display screen, refresh rate of 60 Hz, and were flipped horizontally, so the right lung was positioned on the left side of the screen to replicate the projection radiologist’s view in clinical practice. The participants sat approximately 70 cm away from the screen. At this viewing distance, the images subtended approximately 20° of visual angle and the target subtended approximately 0.8° of visual angle.

For the control participants, experimental sessions took place in a dimly-lit, windowless laboratory at Macquarie University, Sydney. The experiment was presented with MATLAB via PsychToolbox 3 (Kleiner et al., 2007). Stimuli were downsized to 800 × 800 pixels centered on a 1,920 × 1,080 resolution 27-in. Samsung SyncMaster AS950, refresh rate of 120 Hz. The observers sat approximately 70 cm away from the screen. The images subtended approximately 20° of visual angle and the target subtended approximately 0.8° of visual angle.

### Procedure

All participants performed the first four experimental tasks, and 17 of the 26 radiologists participated in the longer version of the study that included the fifth (inverted) task. This task was initially not included due to concerns about time constraints, but was able to be added after early participants were completing the blocks well within the allocated time. All of the control participants performed the five tasks. The tasks were presented in a set order to avoid biasing participants. Participants were asked to look at the cross and press the space bar as soon as a gray dot (low contrast target) appeared. If there was no response, the trial timed out after 4 s. The next trial started with a 100 ms flash of the fixation cross, which remained static throughout the experiment (see Fig. 2). For tasks 2 and 5, the target was shifted 40 pixels in any possible direction from the center of the nodule or the analogous location on the opposite side. The angle of the shift was chosen at random for each trial. In task 1 there were no nodule coordinates, because a normal chest prime was used. The target locations for this task were obtained by pairing each normal chest to an abnormal chest at random, and using the nodule coordinates from the matched pair in the same way as tasks 2 and 5.

A simple detection task avoids high working memory or other tasks demands but it is important to ensure that the participant is not automatically pressing the response button. To reduce the risk of routine responses, we included temporal variability in the target onset. For all tasks (except the nodule detection task) the prime-target stimulus-onset asynchrony (SOA) varied between 400, 416.7, and 450 ms for the radiologists (screen refresh = 60 Hz) and 400, 425, and 441.7 ms for the controls (screen refresh = 120 Hz). The SOAs were selected based on extensive preliminary experiments.^1^ The SOAs for controls were matched as closely as possible to the radiologists’ times, given the different experimental setup. These were randomized within the block. To reduce anticipatory responses, we added catch trials (9%) where no target appeared. If the participants responded to a catch trial they received a red error message on the screen (“Error! No target”). Although the timeframe of our SOAs is within traditional notions of inhibition of return (IOR), it was not feasible to use shorter durations (see footnote 1). The slower timing of our cueing effect relative to typical Posner-type cueing might be due to the cue appearing within a cluttered image rather than in isolation. Data from Donovan and Litchfield (2013) for chest images show slower attentional capture times than those previously reported for mammography, which may also contribute here.

**Fig. 3:**
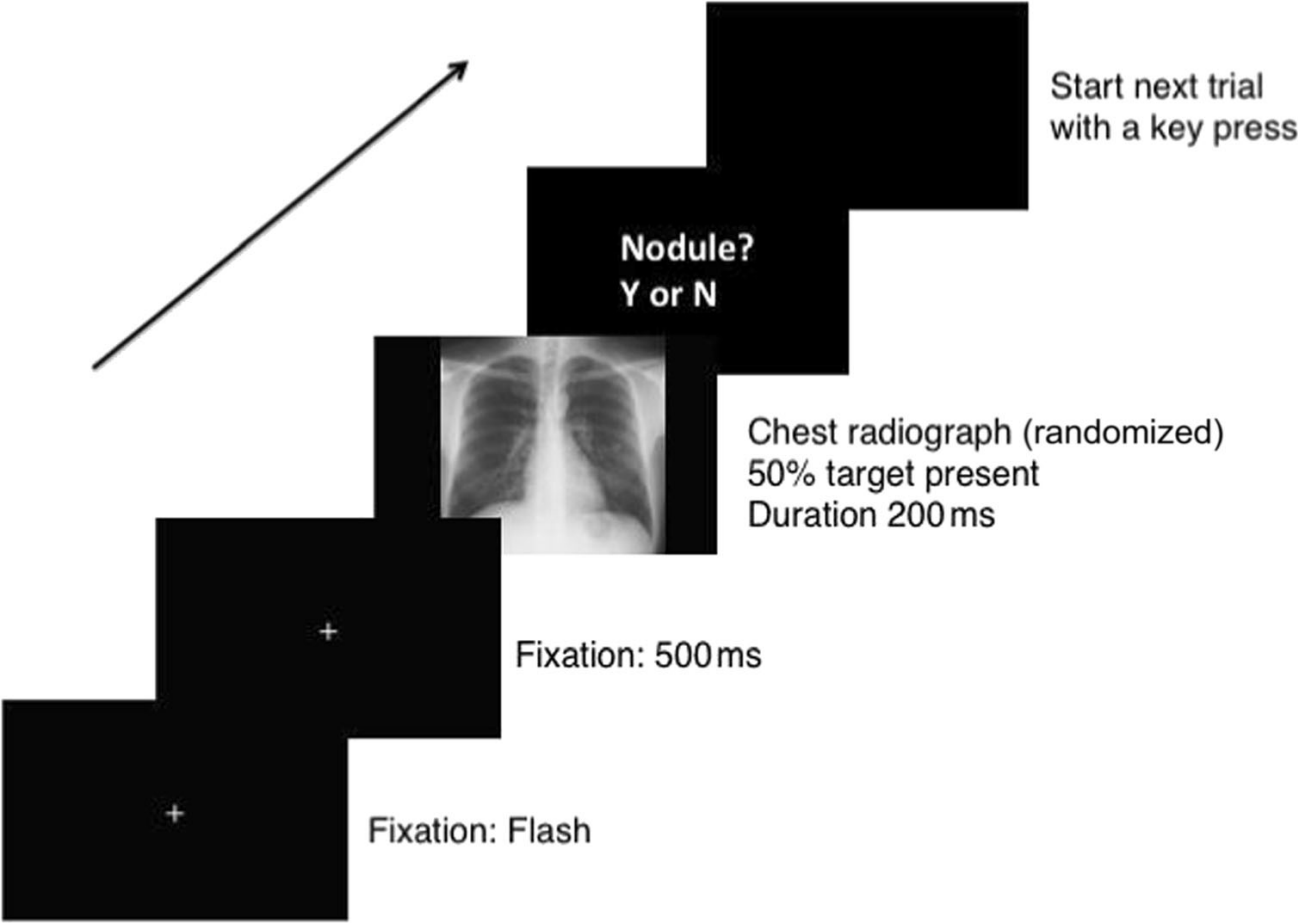
Example of a nodule detection trial shown to the participants

- *Task 1: Chest priors*: On each trial, a normal chest prime was displayed for 200 ms after the fixation screen. Each chest prime appeared on two different trials within the block, one followed by a left target, the other by a right target. The location of the target was based on a 40-pixel shift from an actual nodule location in another image (from Task 2) that was randomly matched to a normal image. The participants saw a practice block of four trials, with images not used in the main experiment, followed by 96 experimental trials, giving a total of 108 trials (including 12 catch trials) resulting in 24 trials/condition (left/right) (Fig. 2a). Apart from feedback when responding during a catch trial, no feedback was provided throughout the experiment.
- *Task 2: Upright nodule*: On each trial, a chest radiograph containing a lateralized nodule (either left or right) appeared for 200 ms after the fixation screen. We constrained target location to match nodule location, with a 40-pixel shift in any direction from the center of the nodule, for each nodule image, creating a valid (same side) and invalid (opposite side) prime-target pair. Observers saw each prime a total of four times: twice in the valid and twice in the invalid condition, randomly intermingled within the block. In total the participants saw 240 trials; four practice trials (two valid, two invalid), with images not used in the main experiment, and 216 experimental trials, giving a total of 48 trials/condition. Breaks were given for 10 s every 48 trials and no feedback was provided. Note: we did not backward mask the stimuli so although timings were precisely programmed, the resulting processing time is only approximate (Fig. 2b).
- *Task 3: Chest priors questionnaire*: The participants were asked to complete a “nodule priors” questionnaire that consisted of a static image of a normal chest radiograph divided into quadrants (see Supplementary Material). First, they were asked to “Please mark 1–4 where you think the likely location for a single pulmonary nodule would occur, with 1 = most likely, 2 = likely, 3 = less likely, 4 = least likely.” Second, we asked, “Do you know the frequencies of nodules in different areas?”
- *Task 4: Nodule detection (present/absent):* We included this condition to test whether the radiologists could discriminate between normal and nodule chest radiographs at the 200 ms presentation duration when it was task-relevant. Both groups performed eight practice trials, with images not used in the main experiment, followed by 96 trials (50% nodule present) where each image from the main experiment (Tasks 1 and 2) was displayed on a black screen (RGB triplet: 0, 0, 0). Note that participants had been exposed to these images in the preceding blocks, making our estimate of their nodule detection likely to be higher than for novel images. Each trial began with a fixation point for 500 ms, followed by a centrally presented chest radiograph (200 ms). After the chest radiograph, we presented a black screen asking the radiologists to categorize the image using a key press to respond to the question “Nodule?”: Y=yes; N=no. The radiologists commenced the next trial with a key press and no feedback was provided (see Fig. 3).
- *Task 5: Inverted nodule:* This task was identical to the upright nodule task except that the chest radiograph primes were rotated 180° (Fig. 2c).

## Results

### Analysis

We calculated mean differences (Mdiff) with 95% confidence intervals (CIs), as well as a Cohen’s *d* estimate of effect size corrected for small sample size, to assist in accurate interpretation of the effects. This latter measure, *dunb,* represents an adjusted, unbiased Cohen’s *d* standardized effect size applied to single sample t-tests where *dunb* = (1 - 3 / (4*df - 1)) * *d* (Cumming, 2012). Exploratory Software for Confidence Intervals (ESCI: Cumming, 2012) and JASP (Version 0.8.6) were used for the analysis.

Frequentist statistics do not allow for the interpretation of null effects – a *p-*value greater than alpha merely informs us that we do not have evidence to reject the null hypothesis. For our analyses we report both frequentist and a Bayes Factor (BF) with a Cauchy prior width of 0.707.^2^

Our dependent variable for target detection was reaction time (RT, ms). Outliers (defined as RTs less than 100 ms and greater than 1,000 ms) were removed prior to statistical analyses. With these criteria, 0.8–1.6% (controls: 0.5–0.7%) of trials were discarded across blocks. Catch-trial errors ranged from 5–11% (controls: 2–5%) across blocks and all were excluded from the analyses. No participants needed to be excluded on the basis of catch trial errors or outlier data.

#### Chest priors

Our first aim was to explore whether there was a cueing effect from chest radiographs without abnormalities due to the evoked context for radiologists.

##### Radiologists

**Fig. 4:**
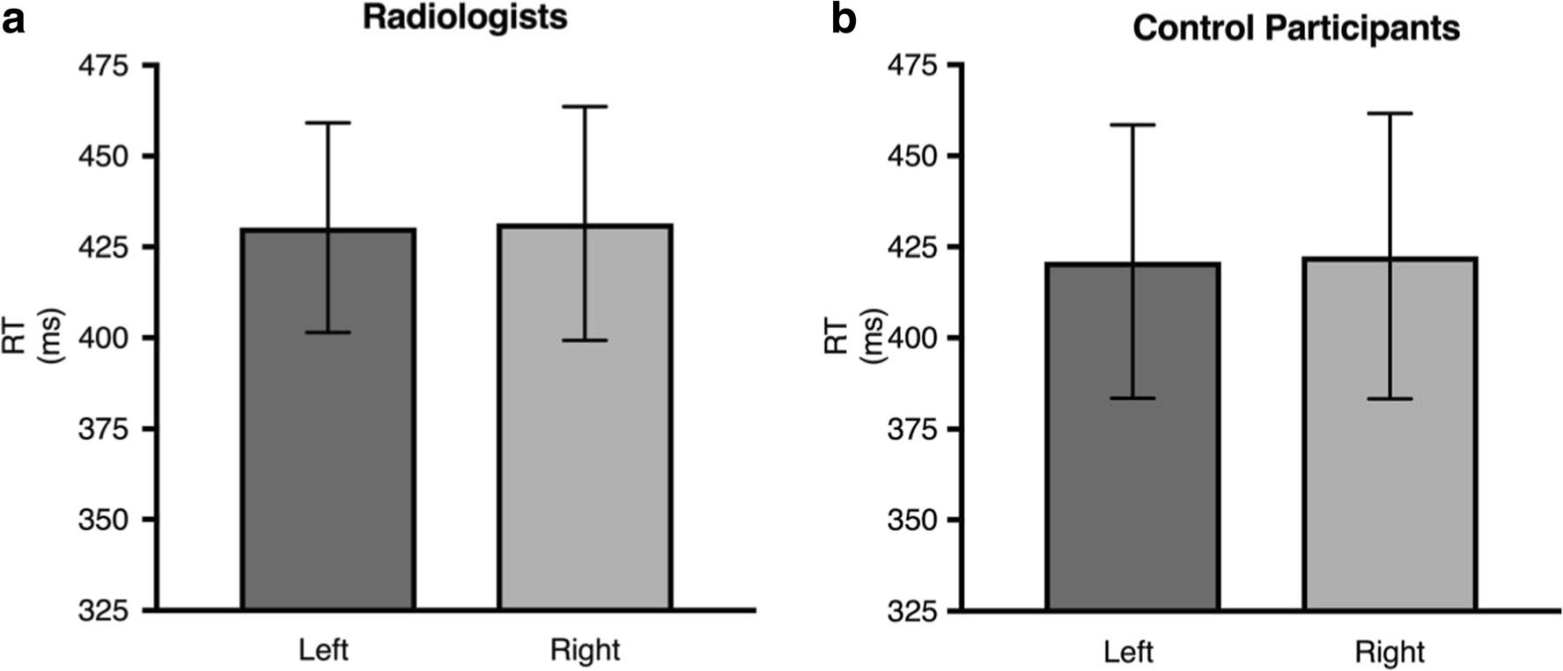
Mean reaction time (RT) for (**a**) radiologists’ and (**b**) control participants’ performance on the chest priors task. The dark gray bars represent the mean RTs for the left-sided target trials and the light gray bars represent the mean RTs for the right-sided target trials. Error bars represent 95% confidence intervals

Lung nodules occur more frequently in the upper right quadrant (Winer-Muram et al., 2002), which is shown in the upper left of the display (radiological convention). Figure 4a shows the mean RTs for trials separated by left-sided targets (dark gray bar) and right-sided targets (light gray bar). A paired-samples *t*-test showed no significant difference in mean reaction time between the left and right targets [*t*(25) = 0.16, *p* = .87, Mdiff = 1.16, CI [-13.54, 15.85], *dunb* = 0.015, *N* = 26, *r* = .89; BF(26) = 0.21]. The BF shows that we have more evidence for the null than for the alternate hypothesis, consistent with the conclusion that a normal chest radiograph does not cause a detectable attentional shift to either the left or the right hemifield.

##### Controls

Figure 4b shows the mean RTs for trials separated by left-sided targets (dark gray bar) and right-sided targets (light gray bar) following a normal chest radiograph prime. A paired-samples *t*-test showed the difference in mean RT between the left and right cued trials was not significant [*t*(25) = 0.205, *p* = .84, Mdiff = 1.5, CI [-13.27, 16.21], *dunb* = 0.015, *N* = 26, *r* = .93; BF(26) = 0.21]. As would be expected, controls do not show attentional shifts from the radiograph prime.

#### Upright nodule and inverted nodule

Our second aim was to explore whether experience captures attention due to higher nodule sensitivity when shown a clinically relevant cue, and if inverting the cues disrupts processing benefits accrued through extensive experience. We therefore analyzed the results of the upright and inverted blocks together, so we can directly test the hypothesis that the pattern is affected by the orientation of the cue image.

##### Radiologists

**Fig. 5:**
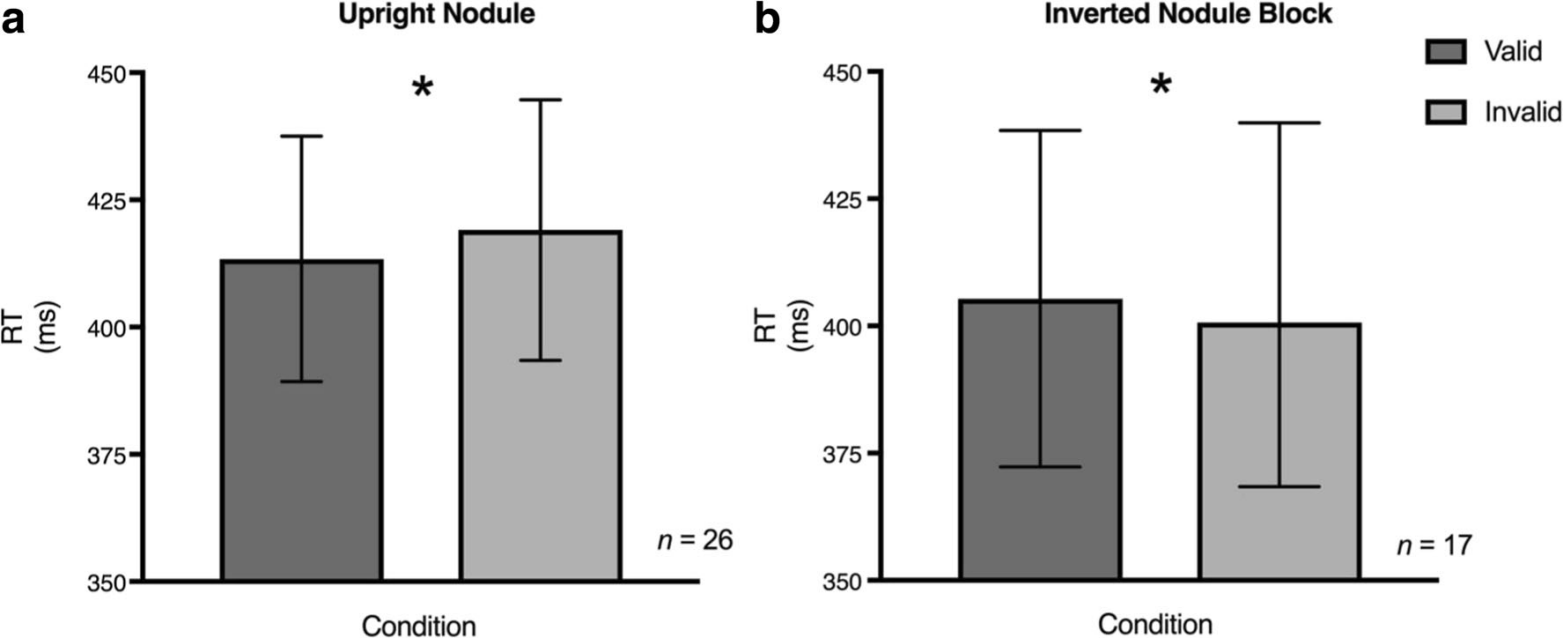
Mean reaction time (RT) for (**a**) 26 radiologists’ performance on the cueing task for the upright nodule task and (**b**) for 17 radiologists’ performance on the cueing task for the inverted nodule task. The dark gray bars represent the mean RTs for the valid target trials and the light gray bars represent the mean RTs for the invalid target trials collapsed across nodule location (left/right). * p<.05. Error bars represent 95% confidence intervals. Note: Validity is collapsed across location

Figure 5 shows the mean RTs for (a) the upright nodule block (n = 26) and (b) the inverted nodule block (n = 17) for valid trials (dark gray bar) where the nodule and the target appeared on the same side compared with the invalid trials (light gray bar) where they appeared on opposite sides. A repeated-measures ANOVA with the factors Orientation (upright/inverted), Location (left/right), and Validity (valid/invalid) showed no main effect for Orientation [*F* (1,16) = 0.82, *p* = .38], for Location [*F* (1,16) = 0.63, *p* = .44] or for Validity [*F* (1,16) = 0.16, *p* = .69]. There was a significant Orientation by Validity interaction [*F* (1,16) = 12.47, *p* = .003, *η*^2^_*p*_ = .44]. *Post hoc* tests on the source of the interaction show for the upright condition the radiologists were significantly faster for the valid compared with the invalid conditions for the upright images [*t*(25) = 2.29 , *p* = .031, Mdiff = 5.67, CI [0.56,12.65], *dunb* = 0.09, *N* = 26; BF(26) = 1.85], and significantly slower for the valid compared with the invalid conditions for the inverted images [*t*(16) = -2.25, *p* = .039, Mdiff = -4.7, CI [-9.14, -0.27], *dunb* = -0.07, *N* = 17; BF(17) = 1.79]. Although the effect sizes are small, this is perhaps not surprising given the task is a simple visual detection. Our BFs are weakly in the direction of the alternative hypothesis. Note: for Fig. 5 validity is collapsed across nodule location.

As we repeated the images within the block, we checked to see whether the repetition of the images had any effect on response time. A paired-samples *t*-test showed that the radiologists were faster for the subsequent than for the first presentation for the valid trials [*t*(25) = -2.21, *p* = .03, Mdiff = -16.26, CI [-31.44, -1.09], *dunb* = -0.25, *N* = 26; BF(26) = 1.61] but there was no difference for the invalid trials [*t*(25) = -1.11, *p* = .28, Mdiff = -11.69, CI [-33.33, 9.96], *dunb* = -0.164, *N* = 26; BF(26) = 0.36].

##### Controls

**Fig 6:**
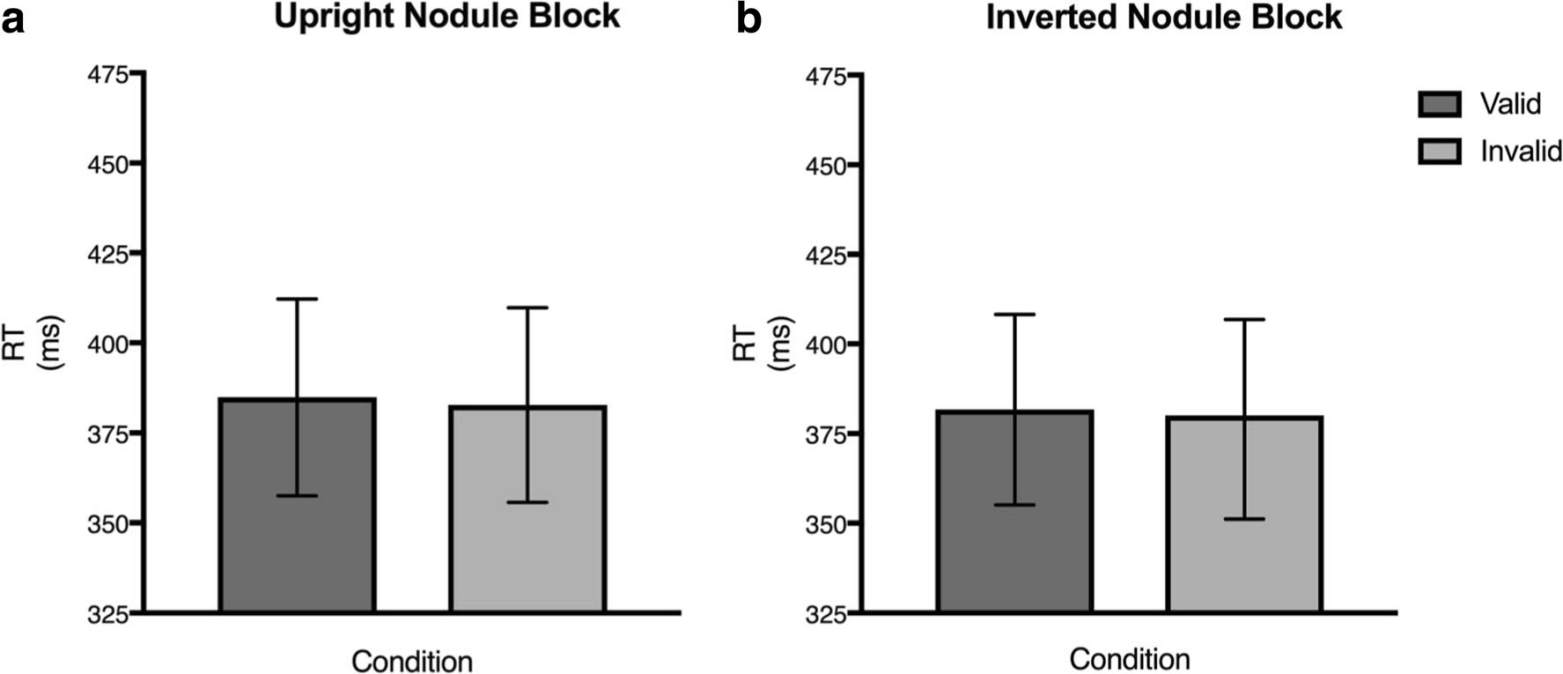
Mean Reaction Time (RT) for the control participants on the cueing task for (**a**) the upright nodule task and (**b**) the inverted nodule task (n = 26). The dark gray bars represent the mean RTs for the valid target trials and the light gray bars represent the mean RTs for the invalid target trials. Error bars represent 95% confidence intervals. Note: Validity is collapsed across location

Figure 6 shows the mean RTs for 26 control participants for (a) the upright nodule block and (b) the inverted nodule block for valid trials (dark gray bar) where the nodule and the target appeared on the same side compared with the invalid trials (light gray bar) where they appeared on opposite sides. A repeated-measures ANOVA with the factors Orientation (upright/inverted), Location (left/right), and Validity (valid/invalid) showed no main effect for Orientation [*F* (1,25) = 0.25, *p* = .62], for Location [*F* (1,25) = 0.03, *p* = .86], and for Validity [*F* (1,25) = 0.98, *p* = .33], and no interactions (*p* > .05). As there is no indication of any interactions, we did not analyze these data further.

**Fig. 7:**
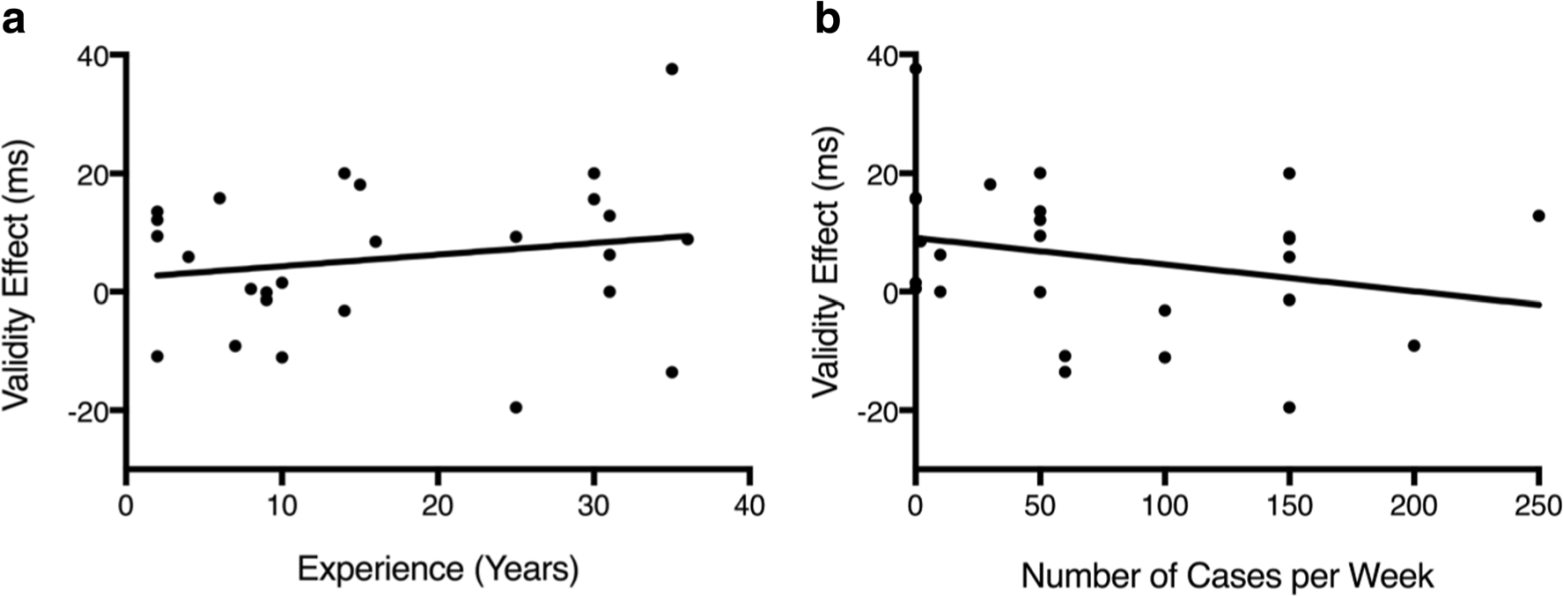
Correlation between the validity effect (y-axis) and (**a**) years of experience and (**b**) number of cases per week (x-axis) for the radiologists

It seems intuitive that expertise would increase with the number of cases and years reading chest radiographs. We therefore conducted a *post hoc* analysis to see if these factors correlate with the cueing effect for the radiologist group. We calculated a validity effect (calculated as a difference score between invalid and valid RT) and correlated years of experience and number of cases read/week, with the magnitude of the attention cueing. There were no significant correlations (Fig. 7; years of experience: [Pearson’s *r*(26) = .19, *p* = .36]; cases per week: [Pearson’s *r*(26) = -.26, *p* = .2]).

#### Chest priors questionnaire

##### Radiologists

Consistent with the actual likelihood, 57.7% of radiologists reported upper right chest as the “most likely” to contain a nodule where chance is 25%. This was followed by the upper left (26.9%), lower right (15.4%), and lower left (0%). Of the radiologists, 23.1% explicitly knew the reported frequencies of nodules in different areas of the lungs; the others reported they did not know.

##### Controls

Only 23.1% of the controls marked the upper right quadrant of the chest as the quadrant most likely to contain a nodule. This was followed by lower right (30.8%), lower left (26.9%), and upper left (19.2%). None of the control participants knew the frequencies of nodules in different areas of the lungs.

#### Nodule detection

We then tested whether the radiologists could detect the nodule when it was task relevant. Note that in our cueing task, the prime itself was completely irrelevant. Here, they were actively searching for the nodule, and they had already been exposed to these images in the previous block, so the estimate of their detection accuracy will be greater than it is likely to have been in the preceding cueing block.

##### Radiologists

Mean percentage accuracy across all trials (nodule present and absent) was 63% (*SD* = 6.76). If we just look at “hit rate,” correct responses for target present trials was 64% (*SD* = 15%). D prime was calculated as a function of abnormality present or absent. Higher *d′* indicates greater sensitivity: the higher the *d′*, the more accurately the radiologists responded to both target present and target absent trials (i.e., reported a nodule when a nodule was present *and* no nodule when no nodule was present). A *d′* of zero indicates there is no sensitivity and the participant is performing at chance (i.e., no better than guessing). Single-sample *t*-tests on average *d′* (0.74) relative to 0 (chance) for nodule detection showed that radiologists do indeed have information about the presence of the nodule at 200-ms duration [*t*(25) = -10.05, *p* < .0001, Mdiff = -0.74, CI [-0.88, -0.58], *dunb* = -2.71, *N* = 26; BF(26) = 3.610e +7].

##### Controls

Mean percentage accuracy across all trials was 54% (*SD* = 5%); controls “hit rate” for target present trials was 49% (*SD* = 11%). Single-sample t-tests on average *d′* (0.26) relative to 0 (chance) for nodule detection showed that control participants have some information about the presence of the nodule at 200 ms duration [*t*(25) = -4.77, *p* < .0001, Mdiff = -0.26, CI [-0.37 -0.15], *dunb* = -0.13, *N* = 26; BF (26) = 372.83].

An independent *t*-test on the mean *d’* values showed that the radiologists performed better than control participants [*t*(50) = -5.17, *p* < .0001, *dunb* = -1.41, *N* = 26; BF(26) = 3671.06]. Thus, although when controls are looking for a nodule they are better than chance, radiologists outperform novices in this task.

**Fig. 8:**
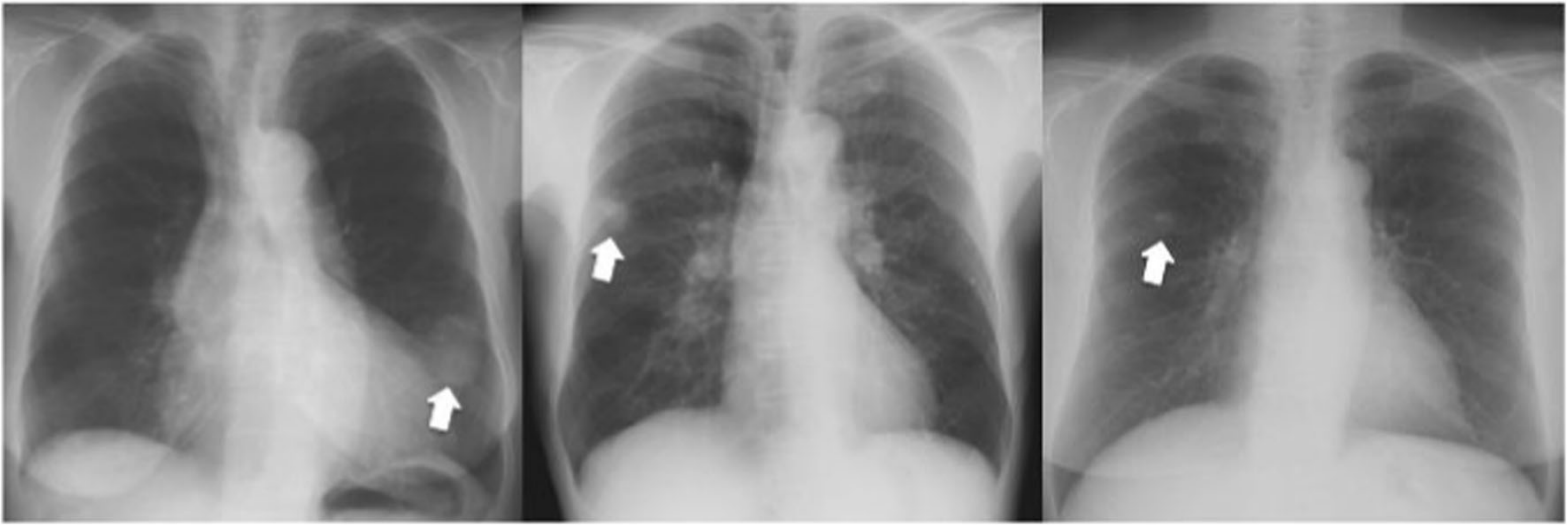
Three images removed *post hoc* where the radiologists were 96–100% accurate on nodule detection. The nodules are indicated by the white arrows (not in actual displays)

As our radiologists’ cueing effect is quite small, it is possible that it is driven by only a few images. Given that real-world stimuli, such as nodules in chest radiographs, have such variability, the characteristics of a few images could result in an apparent effect that might not generalize. We therefore identified the images in which the radiologists performed accurately on the detection task (although note the nodules may not have been obvious in the cueing task as they were task-irrelevant). Figure 8 shows the three images where all of the radiologists (but not the controls) scored the highest nodule detection (96–100%). We removed these images and recalculated upright and inverted cueing means. For the radiologists, a repeated-measures ANOVA with the factors Orientation (upright/inverted), Location (left/right), and Validity (valid/invalid) again showed no main effect for Orientation [*F* (1,16) = 0.8, *p* = .38], for Location [*F* (1,16) = 2.64, *p* = .12] or for Validity [*F* (1,16) = 0.03, *p* = .87]. There was a significant Orientation × Validity interaction [*F* (1,16) = 16.33, *p* = < .001, *η*^2^_*p*_ = .5]. *Post hoc* tests on the source of the interaction show the same pattern as in the original analysis: for the upright condition, radiologists were significantly faster for the valid compared with the invalid conditions [*t*(25) = 2.19 , *p* = .039, Mdiff = 5.52, CI [0.32, 10.73], *dunb* = 0.09, *N* = 26; BF(26) = 1.55], and for the inverted condition, they were significantly slower in the valid compared with the invalid condition [*t*(16) = -2.76, *p* = .01, Mdiff = -5.86, CI [-10.32, -1.41], *dunb* = -0.09, *N* = 17; BF(17) = 4.31]. It is therefore unlikely that the cueing effect for the radiologists is driven by a few images.

## Results summary

Invoking the context of a medical image did not result in an attentional bias to the region of a chest radiograph most likely to contain a nodule. We found a significant cueing effect for radiologists presented with brief chest radiograph primes with a lateralized nodule, despite it being irrelevant to the visual detection task. This occurred in the expected direction for upright primes, and, surprisingly, in the opposite direction for inverted primes. Neither of these effects occurred in naïve controls.

When the chest prime display was relevant, the radiologists were able to detect the nodules that were present at 64% accuracy across the images. We verified that the more obvious nodules were not the only source of the cueing effect.

## Discussion

The successful completion of reading and interpreting medical images by radiologists is crucial for accurate diagnoses and patient care. These complex tasks rely upon the effective engagement of attentional mechanisms. Our first aim was to test whether normal chest radiographs presented as primes would bias radiologists’ attention towards more likely locations for nodules. We found no evidence that the 1.5-fold increase in nodules occurring in the right versus the left lung (Swensen et al., 2000; Winer-Muram et al., 2002) influenced the initial distribution of attention in either radiologists or control participants. Second, we investigated whether nodules in chest radiographs captured attention for radiologists due to their experience. The radiologists showed a significant validity effect; nodules on the same side as a subsequent target resulted in faster detection of the target compared to when the nodules appeared on the opposite side to the target. The BF (1.85) was in the direction of the alternative hypothesis consistent with the frequentist statistics, but the effect was not strong. The validity effect was not present amongst the control observers, suggesting that expertise in reading medical images leads to higher sensitivity to nodules. Surprisingly, when we disrupted processing benefits accrued through extensive experience viewing upright radiographs by inverting these stimuli, we got a reversed cueing effect (invalid < valid) for the radiologists, and again no effect in controls.

We did not find evidence in Task 1 that the statistically more likely (in real-world practice) location of nodules biased the allocation of attention, despite questionnaire data showing some (implicit for most) knowledge of the likely location of nodules. Most radiologists marked the upper right of the chest as that “most likely” to contain a nodule – which *is* the reported most frequent location of primary malignant nodules (Swensen et al., 2000). A small proportion also explicitly knew this information (6/26). The radiologists seem to have implicit knowledge (as indicated by the questionnaire), but this is not automatically activated when seeing chest radiographs. It is possible that we missed an effect because we collapsed across the upper and lower right regions of the chest radiograph as our stimuli did not accurately differentiate this factor. Alternatively, the strength of these learned spatial associations for nodules in a chest radiograph may not be strong enough to influence these initial stages of processing, but might come into play at a later stage of systematic search. Donovan and Litchfield (2013) found that nodule localization in chest radiographs was quite low and time-to-first fixations tended to be much longer for chest radiographs than we see with mammograms. Perhaps these prior probabilities of nodule locations in a chest are not as regular as some other imaging modalities (e.g., Båth et al., 2005) and target location may affect detectability (Håkansson et al., 2005). Last, radiologists are trained to search a chest radiograph in a sequential order and this learned behavior might override the probabilistic bias towards the upper right quadrant. Taken together, we do not see any evidence of a bias towards the most likely location to contain a nodule in the early stages of processing explored in this study, but there might be interesting avenues in examining both different modalities such as mammography, where there are prior probabilities for breast cancer locations, and for examining the effects of prior probabilities on the later stages of search.

A clinically relevant (but task-irrelevant) cue embedded within a chest radiograph captured attention for radiologists but not controls, resulting in a small but significant cueing effect. It has been suggested in the medical perception literature that expertise tunes feature sensitivity (Nodine & Krupinski, 1998). Our results suggest radiologists are more sensitive to relevant clinical features than control participants, which shows up through the nodules in the chest radiograph having a strong enough signal to capture the radiologists’ attention and affect their subsequent behavior. The very same images have no effect on controls, suggesting it is not a simple “bottom-up” salience signal of the nodules that is causing the attention shift. It could be that the radiologists process the whole display faster due to their expertise, similar to scene-based guidance of attention, which is argued to be formed from the image structure and the sum of past experiences (Wolfe et al., 2011). Alternatively (or as well), our effects could reflect a greater sensitivity to the nodule features amongst radiologists, relative to control participants, which results in capture of attention to the location of the nodule. Due to experience, the perceptual features that indicate a nodule might be boosted through perceptual tuning. Kundel and Nodine (1975) showed that when presented with a chest radiograph for 200 ms, experienced radiologists (but not trainees) could detect an abnormality with 70% accuracy. This is consistent with the perceptual tuning hypothesis of expertise where specific features in an image become more salient and overall pattern recognition facilitates diagnosis (Nodine & Krupinski, 1998).

To have sufficient trials with images balanced on nodule location and subtlety, we had to repeat our images. A *post hoc* analysis showed that the radiologists were faster for the trials where they saw repeated images on the valid trials compared with the first presentation. This may suggest an influence of contextual cueing, where the repeated presentation of context either guided attention more effectively (Chun & Jiang, 1998), or it may represent the standard speeding on repetition that is often seen within these types of tasks.

These results provide support for our previous finding (Carrigan et al., 2018) that localization information about an abnormality is available from very brief presentations of medical images. In our study with breast-screening radiologists, we found that presentations of a mammogram of 250 ms were sufficient to support both detection and localization of an abnormal mass. Here, we find evidence for this from a different perspective, exploring indirectly the consequences of expertise and information about mass location on the deployment of attention. Although we do not have information about whether they are explicitly aware of the nodule, our attentional cueing effect demonstrates that radiologists (but not controls) process location information about irrelevant nodules in radiographs presented for just 200 ms.

In addition to the indirect indications of localization information provided by the cueing effect, in the explicit nodule detection task, both groups were above chance. Accuracy was, however, far from ceiling, even for the radiologists (“hit rate” = 64% [*SD* = 15%]; controls “hit rate” = 49% [*SD* = 11%]). The low accuracy in the controls indicates that these nodules were not easy to identify without radiological training, which is consistent with other studies on non-medical participants detecting a mass in a mammogram (e.g., Kunar, Watson, Taylor-Phillips, & Wolska, 2017).

Although *d’* of the control observers was above chance, their average *d’* was still significantly poorer than the that of radiologists (radiologists = .74; controls = .26) and also for what has been reported in free-viewing (where radiologists have *d′* values around 1.0–2.5 (D’Orsi et al., 2013)). Our results are consistent with other medical imaging studies that have shown that radiologists viewing difficult mammograms at 250 ms are able to detect abnormalities at above-chance levels (Carrigan et al. (2018): mean *d′* = 0.5; Evans et al. (2013): mean *d′* = 0.7). In our case, we are probably overestimating the extent to which the nodules were processed in the actual cueing task, as by the time the detection task was completed, participants had already seen the stimuli a number of times and were actively searching for it, whereas in the cueing task the radiograph was novel and irrelevant to the task. Despite this, both groups were still well below ceiling in detecting the nodules. Thus, our cueing effect demonstrates a shift of radiologists’ attention by a task-irrelevant signal that is unlikely to have been consciously detected. Note: the nodule detection task differs from the other tasks in background color. The higher contrast between the black background and the chest radiograph may have reduced visual comfort for the participants, which may have reduced nodule detection accuracy. Alternatively, it may have increased nodule saliency, which may have improved nodule detection. Either way, both the radiologists and the control participants saw the identical tasks (allowing for different testing sites), so we were able to compare their results.

The cueing effect of valid being faster than invalid trials for the nodule condition is consistent with attentional capture by the nodule for radiologists. This was not the case for controls, suggesting that it is not driven by low-level features in the images. The interaction with the inversion condition for the radiologists is driven by a reverse cueing effect (valid *slower* than invalid). We hypothesized that rotating the radiographs 180° (i.e., inverting them) would disrupt experience-based benefits given that the radiologists’ expertise with these stimuli is accrued with them in an upright orientation. Inversion has been shown to disrupt many experience-based perceptual and attentional benefits such as holistic processing of objects of expertise (e.g., Chin et al., 2018; Tanaka & Sengco, 1997), with inverted images often used in control conditions in such studies (e.g., Curby et al., 2009). Inversion has also been shown to reduce the rate of recall in real-world, meaningful scenes, suggesting that semantic memory is needed for scene context (Brockmole & Henderson, 2006). Here we see a reversed cueing effect for the inverted radiographs amongst radiologists, but not controls, suggesting that it is related to their domain-specific expertise rather than lower-level stimulus characteristics. There could be several possibilities for this reversal effect: First, is that we could be seeing an order effect as the inversion task was presented after the upright cueing tasks on the identical images and the questionnaire (which gave clues as to the purpose of the study). This would not have impacted novice performance as they could barely detect the nodules in any case, but this may have had an impact on the experts’ performance. Second, cue processing for the radiologists may have occurred in “radiographic space,” so that when the image was inverted, even if the nodule appeared in the lower left, it would be normalized in the upper right. This is somewhat related to inhibition of return (IOR). According to Posner and Cohen (1984), IOR is observed when attention is withdrawn after being orientated to the cue, which manifests as faster responses in the uncued location. The radiologists may have initially orientated their attention to the cue location, but this was inhibited as a consequence of the image inversion. MacInnes and Klein (2003) studied IOR using complex scenes and proposed that the observed IOR was due to the effect of the visual-motor system “clearing and resetting” data from the visual display. In our study, it may well be that the local cue signal (nodule) was masked by the global signal (whole radiograph) associated with inverting the radiographs. At this point it is not clear what is driving this reverse cueing effect, but importantly it goes in the opposite direction to our key cueing effect from upright radiographs. Further studies are required using inverted images and tightly controlled experimental timings to explore these intriguing findings.

We found no significant correlation between experience and the cueing effect, which could, of course, be a power issue, but could also be due to other influences on expert performance. There is a growing body of literature supporting the notion that experience alone cannot account for all the variation in expert performance. Using tasks that measure domain general visual ability (Novel Object Memory Task (NOMT); Richler, Wilmer, & Gauthier, 2017) and fluid intelligence (Raven’s Advanced Progressive Matrices; Raven, 2000), these factors have been found to account for an additional 15% of variance in a nodule-detection task, over and above radiological experience (Sunday, Donnelly, & Gauthier, 2018). These factors are an important consideration when studying expertise and we are currently exploring these relationships in a medical image context.

A challenge when using medical images is the inherent variability of such stimuli due to anatomic differences across exemplars as well as routine image artefacts. At the outset, we wanted to maintain the ecological validity of the study by showing “true” medical cases rather than artificial nodules. However, such variance in the prime images introduces additional potential noise. For example, distractor features in the images, such as the normal stomach bubble, are present in some of the stimuli, which could attract attention due to the high contrast. However, the current results are useful as they demonstrate that exogenous cueing can occur even when the prime is embedded within a cluttered heterogeneous image, which is quite different from most laboratory cueing studies where factors such as distractors are tightly controlled (e.g., Chun & Jiang, 1998; Posner, 1980). The slower timeframes required to observe such cueing might reflect the additional processing requirements of the cluttered displays. They also suggest that radiologists’ attentional allocation is impacted by images from their domain of expertise, even when these images are irrelevant for their current task. Cues embedded in medical images can drive the allocation of attention for those with experience reading them.

This experiment explores the impact that medical image context can have on the initial deployment of attention. It provides evidence that experts do indeed have their attention captured by information in irrelevant medical images, using a novel cueing paradigm where a chest radiograph served as a prime. We do not see any evidence that attention is shifted by experience about the statistical probability of nodule locations alone, but it is affected by the presence of a subtle nodule in a radiograph. This work suggests that information that is not salient to non-experts affects the attention of radiologists, consistent with the proposal that expertise in reading medical images leads to higher sensitivity to relevant features. The results also provide converging evidence that localization information can be extracted by brief presentations of medical images, which has important theoretical implications in radiology.

## Electronic supplementary material

ESM 1 (DOCX 177 kb)

## Footnotes

1 We ran an initial experiment with novices using simulated obvious nodules to test what SOAs were best for cueing from these images. With timings of less than 300 ms there was no evidence of a cueing effect. We then tested SOAs > 300 ms, again with novices, and now found a cueing effect. We tested this final version of the experiment with real subtle nodules and radiologists in an earlier study (unpublished: Carrigan PhD thesis) and showed a weak effect. The current paper is a full replication of this latter study with new participants.

2 The BF_10_ provides the ratio of the model’s evidence compared to the alternative hypothesis, which assumes that the coefficient for a particular component does not equal 0. We follow Kass and Raftery’s (1995) interpretation such that a BF < 1 indicates that the data support the null rather than the alternative hypothesis, a BF 1–3 indicates *weak* support for the alternative hypothesis (note: Jeffreys (1998) interprets a BF 1–3 as *anecdotal* evidence for the alternative hypothesis; Dienes (2011) suggests this indicates experimental insensitivity), whereas a BF > 3 suggests *strong* evidence for the alternative and a BF > 10 suggests *very strong* evidence for the alternative.
