# Supplementary material for: Exploring the effect of context and expertise on attention: is attention shifted by information in medical images?
Source: Atten Percept Psychophys. 2019 Mar 1;81(5):1283–96. doi: 10.3758/s13414-019-01695-7 (PMC6647457; doi:10.3758/s13414-019-01695-7)
Supplement: Supplementary file 1 — (DOCX 177 kb) [file 13414_2019_1695_MOESM1_ESM.docx]

Participant code _____________________________

This is a posterior-anterior chest radiograph. Please mark 1-4 where you think the likely location for a single pulmonary nodule would occur, with 1 = most likely, 2 = likely, 3 = less likely, 4 = least likely.


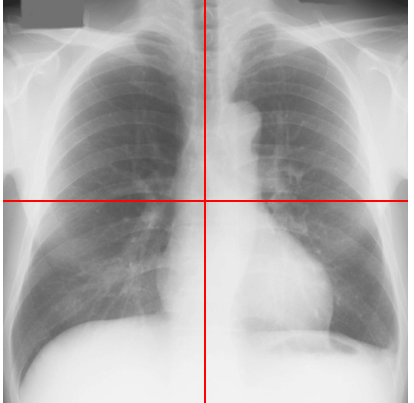


**L**

Do you know the frequencies of nodules in different areas? Yes/No

Thank you for your time ☺
